# New Distributed Fibre Optic 3DSensor with Thermal Self-Compensation System: Design, Research and Field Proof Application Inside Geotechnical Structure

**DOI:** 10.3390/s21155089

**Published:** 2021-07-27

**Authors:** Łukasz Bednarski, Rafał Sieńko, Marcin Grygierek, Tomasz Howiacki

**Affiliations:** 1 Department of Mechanics and Vibroacoustics, Faculty of Mechanical Engineering and Robotics, AGH University of Science and Technology in Krakow, Mickiewicza 30, 30-059 Krakow, Poland; lukaszb@agh.edu.pl; 2 Reinforced Concrete Structures Division, Faculty of Civil Engineering, Cracow University of Technology, Warszawska 24, 31-155 Krakow, Poland; rafal.sienko@pk.edu.pl; 3 Faculty of Civil Engineering, Silesian University of Technology, Akademicka 5, 44-100 Gliwice, Poland; marcin.grygierek@polsl.pl; 4 SHM System Sp. z o.o. Sp. komandytowa., Libertów, ul. Jana Pawła II 82A, 30-444 Krakow, Poland

**Keywords:** distributed fibre optic sensing DFOS, composite, 3DSensor, displacements, settlements, in situ measurements, thermal compensation, embankment, geotechnics, laboratory

## Abstract

Thanks to the dynamic development of advanced building technologies as well as the growing awareness, experience and responsibilities of engineers, structural health monitoring systems (SHM) are increasingly applied in civil engineering and geotechnical applications. This is also facilitated by the construction law and standard requirements, e.g., the observation method for geotechnical structures described in the Eurocode 7. Still, the most common approach is to apply spot sensors in selected points of the structure to validate theoretical models, numerical simulations and support technical assessments by involving statistic and approximation methods. The main limitation of spot sensing is the inability to detect localized damages such as cracks, fractures, sinkholes or shear planes. Thus, such analysis is subject to considerable uncertainty, especially within geotechnical structures, characterized by random mechanical parameters that change with location, but also over time. Another approach is based on distributed fibre optic sensors (DFOS), which are finding a growing acceptance in laboratory and field projects, overcoming limitations of conventional measurements. The design and applications of new DFOS dedicated for 3D displacement sensing are described hereafter in the article. The novelty of the presented solution lies in several features, including design, application, production technology and materials. This article is focused on the operational rules governing DFOS and proving their effectiveness in laboratory and geotechnical field applications.

## 1. Introduction

### 1.1. General Background

Modern civil engineering is not only about creating unique building objects, but above all about using new materials, construction solutions and improving existing technologies. The optimization process should provide both financial savings as well as an appropriate and acceptable level of safety, expressed by reasonably low failure probability and risk [1]. Optimal decision making [2] is a challenging task, not only while managing the construction site at the initial stage on investment, but also through the entire life cycle of the structure [3]. This simple insight is reflected in recommendations included in the basic European standard for structural design EN-1990 [4], where control procedures relevant to the particular project must be specified for design, production, execution and further operation of the structure.

One of the present-day means of effective control is the application of well-designed structural health monitoring (SHM) systems [5,6], of which the main aim is to provide reliable data related to the technical condition of the structure over time [7]. Such systems were originally developed and used in the aerospace industry [8] to identify threats, weaknesses and thus support safe, mass-produced aircraft and space technologies. Particular attention was paid to distributed systems based on optical solutions [9,10], which were able to measure selected physical quantities along the entire length of the linear sensor, while not interacting in any way with electrical wires or devices. Thanks to this approach, it was possible to create a complex system of sensors integrated with the monitored structure and operating in a manner similar to the nervous system of the human body [11,12].

Distributed fibre optic sensing (DFOS) is now also used widely in many other technical sectors, including civil engineering [13] and geotechnics [14]. It overcomes many limitations of conventional spot techniques (e.g., inductive, vibrating wire [15,16] or fibre Bragg gratings [17]), which are unable to detect localized damages such as cracks and fractures in concrete or sinkholes and shear planes in ground structures. Data analysis from spot sensors is related to high uncertainties, especially for heterogenous materials and structures (such as in geotechnics) due to the randomness of mechanical and physical parameters, which can vary not only over time, but also by location. Usually, reliability assessment is supported by finite element analysis (FEA) [18] and advanced numerical models created in a three-dimensional space. However, many theoretical assumptions and engineering simplifications must be applied and their validation by data in single points is subject to a high risk of error or misinterpretation.

**Figure 1:**
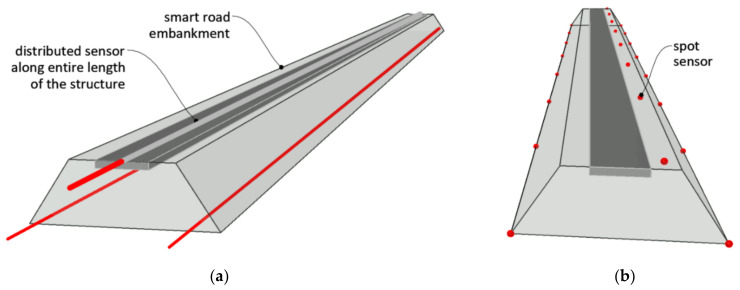
Idea of embankment settlements measurements using: (**a**) distributed technique; (**b**) spot technique.

Much more comprehensive data can be gathered by DFOS sensors, which measure the parameters continuously over the entire length of optical fibre (Figure 1a), replacing thousands of conventional spot-type sensors (Figure 1b). This is a new quality in structural measurements and the reason why DFOS sensors are particularly useful for monitoring linear structures such as roads, highways, embankments, pipelines, tunnels, dams or hydrotechnical structures.

In the example section of embankment in Figure 1, which is 100 m long, three distributed sensors provide 30,000 measuring points (with spatial resolutions equal to 10 mm), while in the conventional approach, there are only several spot sensors or geodetic benchmarks. This enormous difference in the quantity and quality of obtained data is the reason why distributed sensing is nowadays finding a growing acceptance in laboratory, but above all, in field applications. What is more, it is possible to integrate linear DFOS sensors inside the structure during its construction [19,20,21] (or the production of precast structural members), which brings many benefits, including:

- The possibility of analysing the structural behaviour from a real zero state, which is impossible with installed sensors within existing structures with unknown initial levels of stress and or/deformation;
- Integration inside the structure (ground or concrete) providing the more accurate transfer of the measured physical quantity from the structure to the sensor—no additional mounting brackets or installation methods are needed;
- Natural and effective protection of the sensors integrated inside the structure against mechanical damages or harsh environmental conditions. The predicted operation lifetime of such a system is comparable with the operation lifetime of the structure itself.

It is also worth underlining that the presented DFOS approach is in line with the observational method [22] presented in the European geotechnical standard EN 1997 (Eurocode 7). This method relies on the design improvements based on observations made during construction. The efficiency of the method lies in “reliable data of appropriate type or in sufficient quantity”.

### 1.2. Distributed Sensing Principles and Sensors

**Figure 2:**
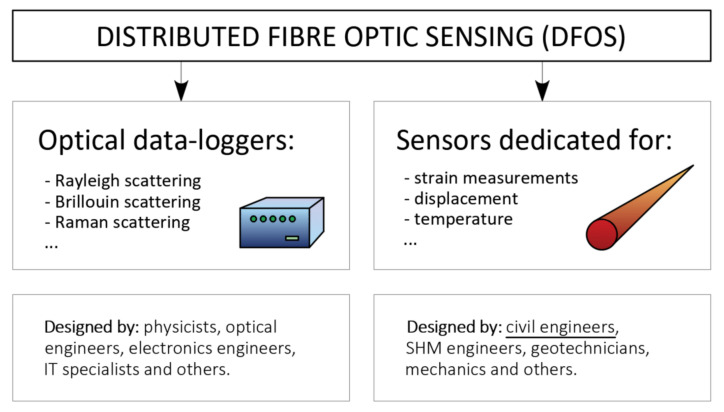
Scheme of distributed fibre optic sensing (DFOS) components within civil engineering.

Distributed fibre optic sensing consists of two areas whose properties determine the efficiency and functionality of the entire monitoring system—Figure 2. The first area is related to optical data-loggers (e.g., reflectometers or interrogators), which allow one to perform measurements of selected physical quantities (usually strains or temperatures [23]). The basic measurement principles involve light scattering: Rayleigh [24], Brillouin [25,26,27] or Raman [28]. Each technique is characterized by its own advantages and limitations, and the final selection should depend on the requirements of a given project. For instance, the Rayleigh-based system provides extremely high spatial resolution [29,30] starting from as fine a width as 5 mm, which allows for the detection of localized damages [31] such as cracks within concrete structures [32]. What is more, dynamic, real-time measurements are also possible [33] with a spatial resolution starting from 0.6 mm. On the other hand, Brillouin systems enable measurements over very long distances (up to hundreds of kilometres) to monitor linear structures and identify extensive damages, such as leakages in hydrotechnical structures, including pipelines.

Optical devices are usually designed and constructed by physicists, optical and electronics engineers as well as IT specialists. However, design of the sensors requires the knowledge provided by civil engineers, geotechnical engineers and mechanics. As the sensor becomes an integral part of the structure, its construction has direct influence on the strain transfer mechanism, [34,35] and thus on the final accuracy and data reliability.

The great majority of the in situ installations described in the literature are related to applications of sensors dedicated for axial strain measurements [36,37]. The main task of such sensors is to provide accurate strain transfer from the monitored structure to the optical fibre located inside the sensor body. It can be provided by a monolithic, composite cross-section with a large range of elastic behaviour, while layered solutions with steel and plastic elements are not adequate due to their yielding and debonding effects [38].

**Figure 3:**
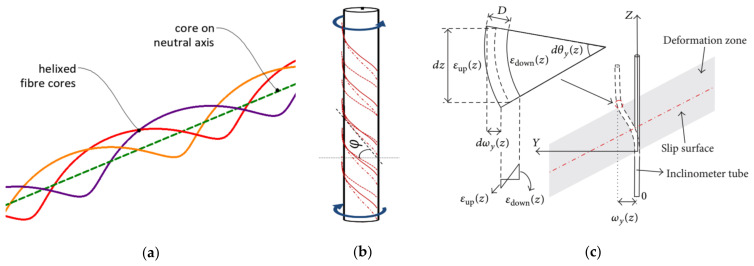
Example optical solutions for displacement measurements: (**a**) helical multicore fibre; (**b**) set of three standard optical fibres in appropriate arrangement [41]; (**c**) mathematical model illustrating the principle of fibre optic inclinometer for horizontal displacements [42].

**Figure 4:**
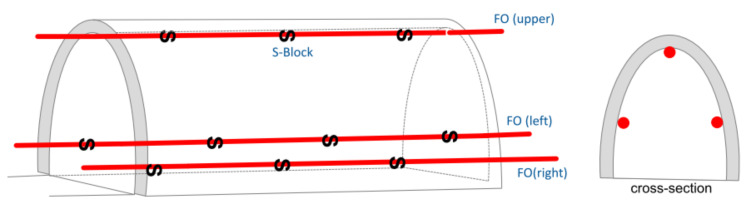
Example layout of DFOS strain sensors for displacement calculations of tunnel lining (for 3D calculations, at least three fibres are required) [41].

Another group of solutions are those dedicated to displacement measurements. According to the state-of-the-art review and authors’ own experiences, there are no sensors dedicated strictly for measuring displacements within civil and geotechnical structures. Previous attempts to analyse shape changes using distributed fibre optic sensing were of a scientific nature rather than an engineering exercise, i.e., focusing on wide practical applications. Several possibilities for shape and displacement measurements were analysed, e.g., using:

1. Helical multicore optical fibres instead of a standard single core—Figure 3a [39,40];
2. Rod with spiral optical fibre arrangement—Figure 3b [41];
3. Mathematical–physical models [42,43] (Figure 3c), allowing one to calculate displacements based on the results from appropriate arranged strain sensors–Figure 4 [41].

The above-mentioned propositions in the form of multicore fibres or spiral single-core fibres did not find any wide uses in the construction sector due to their lack of appropriate protection against harsh environmental impacts, high production costs and other technological difficulties related to the precision of manufacturing. Their applications are limited to laboratory conditions [39,41]. Furthermore, the obtained accuracy is often not acceptable. Previous calculations [39] took into account vertical displacements (deflections) of flexible structures similar to aeroplane wings based on data obtained by surface-installed helical multicore fibres. During one of the load steps, the root mean square error was equal to 6.5 cm, which corresponds to 4.6% of the maximum displacement value (1.4 m). During the entire research, the averaged RMS error was 5.8 cm, while the maximum was 14.1 cm [39]. Analysis was performed in laboratory conditions and only over a short length of 10 m.

The third approach based on analytical calculations involving measured strain values can be used successfully, but only under strictly controlled circumstances, e.g.,

- The linear and elastic behaviour of the structure;
- Homogeneous materials without any discontinuities such as cracks or fractures;
- Accurate strain transfer from the structure to the fibre;
- Appropriate thermal compensation;
- The sufficient precision of sensors’ positioning in field conditions.

The above circumstances significantly limit the effective applications of mathematical models for displacement calculations. They are the reason why very often the obtained accuracy is not acceptable. However, they can be used as a guiding principle for the construction of the new DFOS sensor, which is described hereafter in the article.

The novelty of the presented solution lies in several features, including the **special design** for 3D displacement measurements, **new applications** in civil engineering and geotechnics, as well as the production technology and the material which makes up the sensor core. The purpose of the study is to present and discuss the operating principle (**design**) as well as case study (**application**) related to geotechnical structure (road embankment).

The presented research proved the high-quality performance of the sensor under harsh environmental conditions as well as the efficiency of its thermal compensation system. Example results verified by a number of reference techniques, including the inclinometer system, are presented and discussed hereafter, indicating advantages of this solution for possible structural health monitoring.

## 2. New Distributed Fibre Optic Displacement Sensor

### 2.1. Basic Concept and Design Stage

The idea of a new displacement sensor (hereafter called 3DSensor) for new applications in civil engineering and geotechnics lies in the use of standard single-mode fibres known from a wide range of telecom applications. Such fibres can be used for DFOS strain measurements. They are compatible with most optical devices (data-loggers) and thanks to their widespread use, they are commonly available and cheap. The fibres must be precisely arranged around a main core of the sensor, which should be made of material characterized by a high elastic range and low elastic modulus. This range ensures the proper performance of the fibres, avoiding plastic effects such yielding or debonding from the core. Sensors are dedicated to work in difficult conditions, where localized events can appear, e.g., ground fractures or shear planes. A high elastic range will prevent the sensors from breaking during a long-term operation and reflect the actual state of the structure.

**Figure 5:**
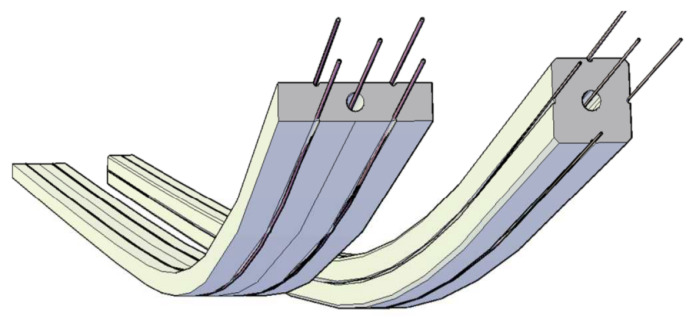
Initial concepts of 3DSensor consisted of a composite core and a set of precisely arranged optical fibres for strain measurements.

Optical fibres must be integrated with the core in a very precise way, as this precision considerably influences the final accuracy of the sensor. Manufacturing by hand is not sufficient, so an automated production line had to be developed to ensure the sensor’s appropriate parameters. The initial concept of the 3DSensor consisting of the core with precisely arranged optical fibres is presented in Figure 5.

For calculation displacements in three-dimensional space, at least three optical fibres are required. However, to apply a statistical approach to improve the accuracy and reliability of the entire system, one additional fibre is used. The calculated displacements depend not only on strains generated in fibres by changes in the sensors’ shape, but also on geometrical dimensions of the core as well as boundary conditions (e.g., cantilever static scheme with fixed rotations and movements at the start point of the sensor):

(1) $$d\left(x\right)=f\left(\varepsilon_{1}\left(x\right),\;\varepsilon_{2}\left(x\right),\;\varepsilon_{3}\left(x\right),\;\varepsilon_{4}\left(x\right),\;b,\;h,\;bc\right)$$

where *ε*_1_, *ε*_2_, *ε*_3_, *ε*_4_ are the strain measured by the fibres, *b*—width of the core, *h*—height of the core, *bc*—boundary conditions.

Despite the above mathematical idea being relatively easy and described before in the literature [43,44], attempts to implement it in practice have never resulted in a ready-to-use solution with documented scientific research and a range of proven field installations. Achieving the high precision in the fibres’ arrangement was possible thanks to the construction of an advanced technological line, allowing the production of continuous composite elements in a pultrusion process. The composite core of the sensor is characterized by a very low elastic modulus (≈3 GPa) and very high strain range (±4%).

### 2.2. Data Processing

**Figure 6:**
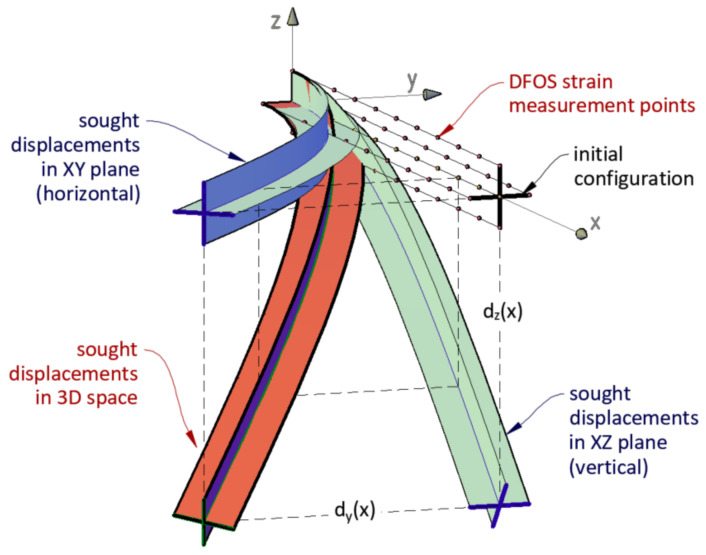
Definition of mathematical–physical problem for calculating 3D displacements based on strain data measured by 4 optical fibres with defined spatial resolution.

The measurement problem is represented by the physical model shown in Figure 6. Strains are measured by four optical fibres with a defined spatial resolution depending on the optical data-logger applied. With the known and constant geometry of the sensor, as well as the known boundary conditions (fixed start point), horizontal and vertical displacements are determined. In its simplest terms, spatial displacements can be expressed as the sum of displacements in both planes.

During research, four algorithms for data processing were considered and/or elaborated. The simplest was based on the beam deflection equation known from structural design (double integration of the curvature). However, this approach is characterized by the lower accuracy confirmed by laboratory studies, as well as only being correct in the range of small displacements, where geometrical nonlinearities can be neglected. Two others are based on solving a system of differential equations (explicit methods of the first and the second order). They are characterized by very good accuracy, but they require advanced mathematical software and are not suitable for engineers in their daily practice. The last method is the most intuitive one, with relatively simple engineering interpretation. It is based on geometrical relationships discussed hereafter. Its accuracy was proved at the same level as in the case of explicit methods by means of a number of numerical simulations. That is why it was finally chosen for data processing.

**Figure 7:**
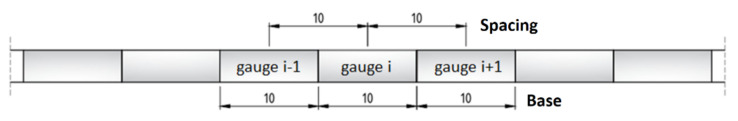
Parameters of distributed fibre optic strain measurements (both gauge’s spacing and measuring base set to 10 mm as an example) [45].

The following assumptions and simplifications were adopted in the algorithm:

1. Sensor’s core works in the elastic range of strains;
2. The Euler–Bernoulli hypotheses that plane sections remain plane and normal to the axis of the sensor’s core;
3. The geometry of the core (height and width) and resulting distance between the opposite optical fibres, are constant over length;
4. Production tolerances are known (e.g., standard deviation of the height over length) and can be used to assess the final accuracy;
5. The superposition principle applies when calculating displacements in 3D space (displacements could be calculated separately for vertical XZ and horizontal plane XY);
6. Boundary conditions are known (rotations and displacements in at least one measuring point or displacements in at least two measuring points);
7. Strains are measured with defined spatial resolution *r* (spacing) and with known accuracy provided by the calibration process;
8. Strains are averaged over the gauge length equal to the spatial resolution, so that the series of front-connected gauges is created—Figure 7;
9. Displacements are calculated in the same fixed points defined by spatial resolution such as in the case of strain measurements (values are linearly interpolated between the points in their simplest terms).

**Figure 8:**
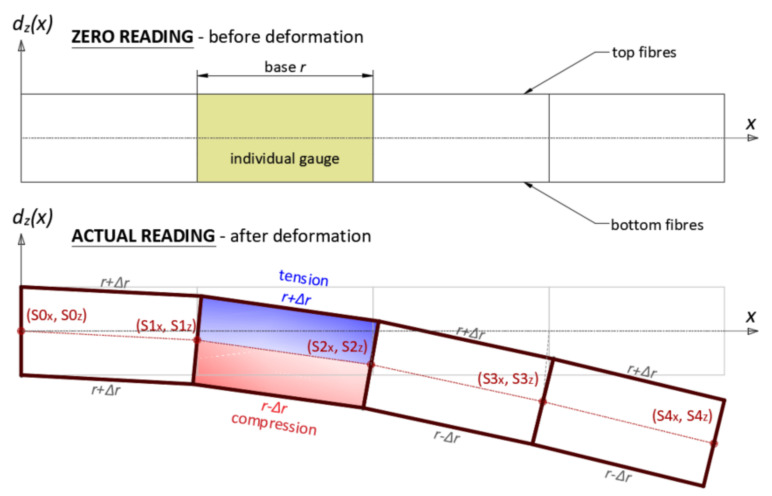
Discretization of 3DSensor for displacement calculations using trapezoid method.

The above method is based on the discretization of the sensor according to applied spatial resolution, creating the chains of individual gauges, which are rectangular at initial configuration (during zero reading)—Figure 8. For further clarity of presentation, analysis is be presented only for the vertical plane XZ, involving the bottom and top optical fibres. Data processing in the horizontal plane XY is analogous, but based on the right and left fibres.

Deformation related the change in the shape of the sensor generates strains within the bottom and top optical fibres. Assuming a statically determined scheme and no presence of axial effects, the absolute values in tension and compression at opposite surfaces of the sensor core will be equal. The influence of axial effects, including force or temperature, are discussed in Section 2.3.

**Figure 9:**
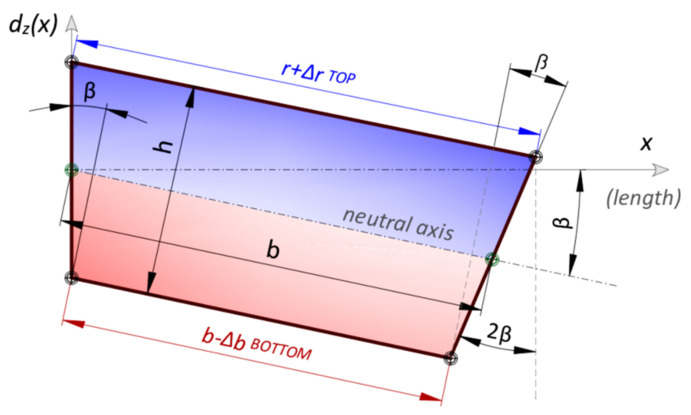
Individual, deformed gauge represented by trapezoid (adapted from [46]).

The rectangular shape of the gauges after deformation are changed into trapezoid (Figure 9) and further analysis is focused on its geometry. That is why the proposed method is also called the *trapezoidal method*. Elongations or shortenings of the gauge bases are calculated knowing the measured strains in optical fibres and using the engineering strain definition:

(2) $$\Delta r_{i}=\varepsilon_{i}\left(x\right)\cdot r$$

To improve accuracy, strain values are averaged from two top and two bottom fibres. The sensitivity of the sensor to displacement changes can be improved by increasing the geometrical dimensions. The greater the core height, the further the fibre from its neutral axis and the greater the measured strains for the same vertical displacements. It is an important feature allowing for the adjustment of the sensor’s parameters depending on predicted displacements in a given project.

Once the geometry of an individual trapezoid (*i*) has been unambiguously defined, its rotation *β* in space is determined by the deformation of the previous gauge (*i*−1). Individual displacements *d_i_* are summed over their entire length *L* to produce the desired displacement profile (*N*—number of gauges over length):

(3) $$\Delta_{tot,i}=\overset{i=N}{\underset{i=1}{\sum }}d_{i}$$

### 2.3. Thermal Self-Compensation System

**Figure 10:**
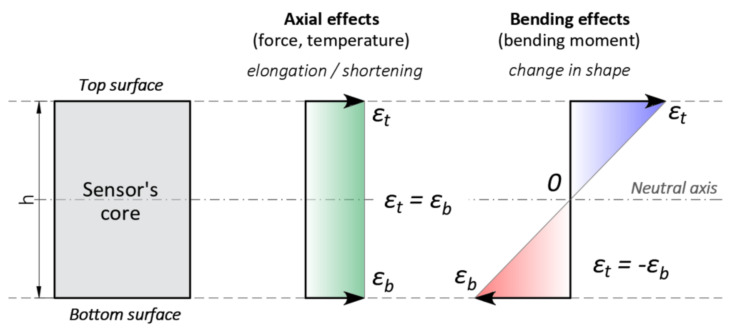
Individual, deformed gauge along the sensor represented by a trapezoid.

The novelty lies in the ability to measure displacements in three directions: X, Y and Z. The concept of the sensor involves the application of additional fibre located freely inside the core close to its neutral axis X. Such location minimizes all influences related to bending (theoretical strain value is equal to 0—Figure 10) and thus allows for measurements of temperature changes over the length. What is more, by using Raman-based distributed optical reflectometers, the absolute temperature of the core can be determined. Based on this data, it is possible to compensate axial shortenings or elongations along the sensor (X direction), as well as perform analysis of structural behaviour subjected to thermal influences.

Despite the application of an additional compensating fibre, it should be noted that the proposed *trapezoidal method,* based on elongations and shortenings within the opposite surfaces of the core, provides thermal self-compensation for displacements calculated in Y and Z directions (plane XY and XZ—Figure 6). The only requirement for correct performance is that temperature changes should be uniform within the entire height of the sensor. Such conditions are provided by low (mm-order) core dimensions and an external coating, which is also used as mechanical protection.

Calculated displacements in Y and Z directions are only caused by bending effects, while axial forces or uniform temperature changes cause elongations or shortenings along the core (X) without changing the sensor’s shape (the same strains in the top and bottom surface of the core). This leads to the conclusion that contrary to common DFOS strain sensors, thermal compensation of the proposed solution is not necessary because displacements are calculated correctly regardless of temperature changes. This note applies to displacements in plane XY and XZ. However, for the correct analysis of 3D displacements (including X axis), additional compensating fibre is required.

**Figure 11:**
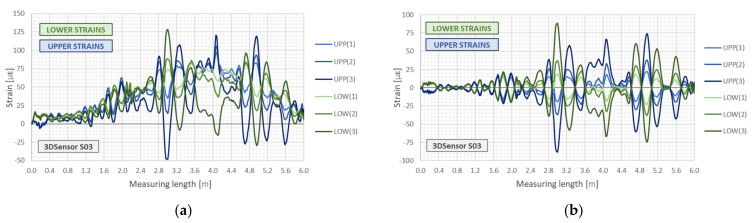
Example strain data from lower and upper fibres in the 3DSensor core for selected load steps 1–3: (**a**) raw data including the bending and the axial effects (force and temperature) [46]; (**b**) compensated due to the axial effects (including only bending) [46].

Having strain measurements on both sides of neutral axis, it is possible to distinguish between axial and bending effects and remove the constant (axial) part. The example of such analysis is presented in Figure 11 based on the practical application of 3DSensor in geotechnical research [46]. Figure 11a shows raw strain data from both lower and upper fibres of 3DSensor at selected load stages, without any compensation. Raw data are influenced by bending, the axial force and thermal actions. On the other hand, Figure 11b shows strains resulting only from bending, where axial actions are removed. Absolute values on both sides are now consistent and correspond to theoretical predictions (Figure 10).

### 2.4. Laboratory Issues

Although the general operation principle of the presented solution seems straightforward, the final success understood as the possibility of widespread use in practice, was possible only by solving a number of issues and technical details. Thus far, these details have been a limitation to the feasibility of developing a DFOS-based displacement sensor for civil engineering and geotechnical applications. The performed research involved theoretical studies (both analytical and numerical simulations), laboratory tests and several field applications. Lessons learned during this process are briefly summarized in Table 1 to illustrate the complexity of the issue under consideration.

### 2.5. Ready-to-Use Sensors

**Figure 12:**
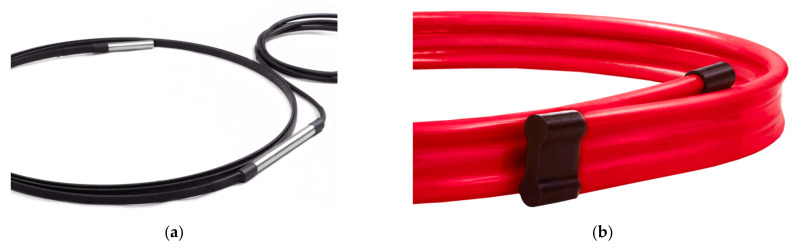
Composite DFOS fibre optic 3DSensor for displacement (shape) monitoring: (**a**) laboratory version; (**b**) in situ version for field applications.

The final step of the research process was to physically create the sensors, which can be ready-to-use in real case studies, both in the laboratory and field conditions. The task was accomplished by setting up a production line in which optical fibres are permanently and precisely integrated into the composite core during the pultrusion process [47]. The core of the sensor has a very low elastic modulus (≈3 GPa) and very high strain range (±4%). The standard dimensions of the field version of 3DSensor are 50 × 15 mm. However, the geometry and physical properties of the senor’s core can be freely modified to meet the requirements of specific projects. Two typical versions were proposed and produced:

1. **Laboratory** with reduced cross-sectional dimensions and stiffness, as well as soft external protective coating—Figure 12a;
2. **Field** with increased cross-sectional dimensions and stiffness, as well as robust external protective coating—Figure 12b.

The prototype version of the sensor, which was ultimately demonstrated within several field installations (including bridges, geotechnical structures and road embankments, described in Section 4), consisted of a highly elastic composite core, optical fibres, appropriate external coating and connections with the pigtails.

### 2.6. Data Acquisition Systems

The application of standard single-mode fibre SM9/125 allows for its use with most of the optical data-loggers available on the market. However, during all laboratory and field studies described hereafter in the article, optical backscatter reflectometer OBR 4600 [48] produced by Luna Innovations was applied. Selected measuring parameters required for correct data interpretation are summarized in Table 2. As there are a number of optical fibres inside one 3DSensor, as well as due to the projects with several 3DSensors installed at the same time, the measurements were performed using an optical switch to speed up and streamline the works.

## 3. Laboratory Studies

This section is focused on two examples of laboratory investigations, involving different static schemes and lengths of prototype versions of 3DSensor, which were carried out before installation in field conditions. It should be emphasized that the research described hereafter is only a short extract from the entire studies, which covered much broader issues presented briefly in Table 1.

### 3.1. Cantilever Scheme

**Figure 13:**
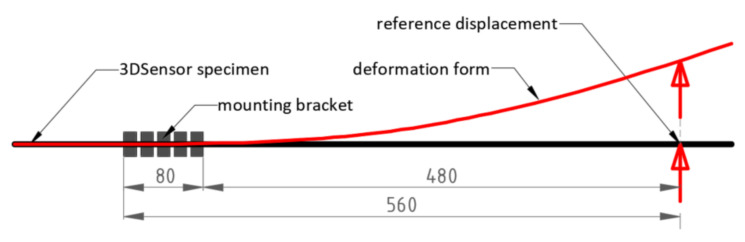
Static scheme of the analysed short-length 3DSensor.

**Figure 14:**
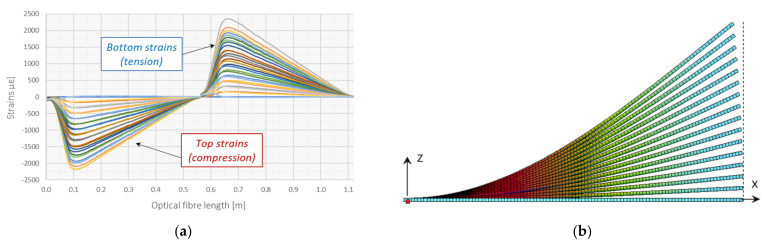
(**a**) Raw strain data during subsequent load steps; (**b**) visualization of deformed FE model used for comparison.

The first test involved short-length sensor specimens analysed in a cantilever scheme—Figure 13. One optical fibre was installed within the lower (tensile) and the upper (compressed) surface, so that the raw strain data are presented within the single plot—Figure 14a. Displacements were applied using a screw mechanism, which was controlled through electronic micrometre with the measurement resolution of ±0.001 mm and accuracy of ±0.005. Reference micrometres were used for error analysis while calculating vertical displacements. However, the nonlinear effect of shortening the specimen over the horizontal axis *x* was also investigated and compared to the results from finite element analysis (FEA). The model (Figure 14b) took into account nonlinear behaviour of the element by applying third order theory.

**Figure 15:**
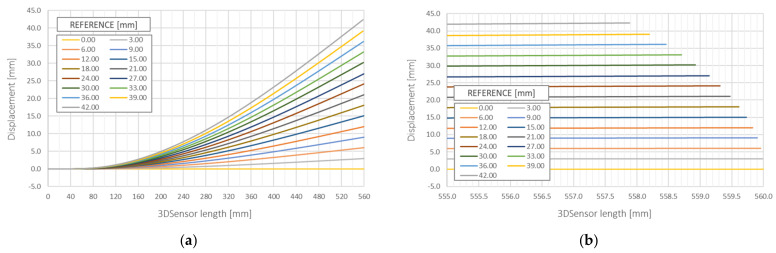
Measurement results: (**a**) vertical displacements in full scale of horizontal axis; (**b**) close up to the end of the specimen with visible shortenings along horizontal axis caused by large vertical displacements (compare Figure 14b).

**Figure 16:**
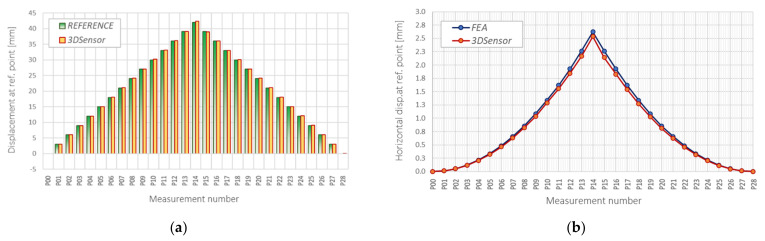
(**a**) Comparison of DFOS vertical displacements with reference micrometres; (**b**) comparison of DFOS horizontal shortenings with FEA results (see also Figure 1b and Figure 15b).

Based on strain data obtained with 10 mm spatial resolution, vertical displacements (Figure 15a), as well as horizontal shortenings over *x* axis (Figure 15b) were calculated for different values of vertical displacements (up to 42 mm). The obtained results are summarized in Table 3 with comparison to reference techniques:

1. Micrometres for vertical displacements (Figure 16a); mean absolute and relative errors: 0.077 mm and 0.427%; corresponding standard deviations: 0.076 mm and 0.424%;
2. FE analysis for horizontal shortenings (Figure 16b); mean absolute and relative errors: 0.042 mm and 4.707%; corresponding standard deviations: 0.035 mm and 1.049%.

The absolute and relative errors *e* presented in Table 3 for subsequent load steps were calculated based on reference readings *d_ref_* using the following equation:

(4) $$e_{relative}=\frac{e_{absolute}}{d_{ref}}\cdot \;100\%=\frac{d_{ref}-d_{3DSensor}}{d_{ref}}\cdot \;100\%$$

### 3.2. Simply Supported and Continuous Beam Scheme

**Figure 17:**
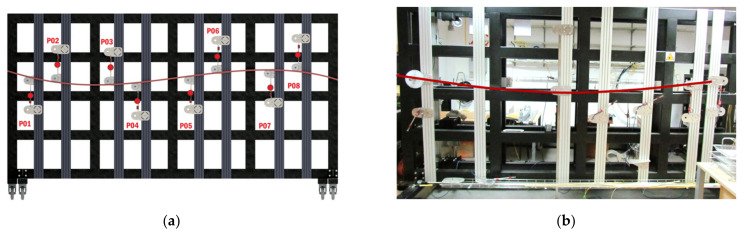
Steel frame as a part of measurement station: (**a**) design; (**b**) view in the laboratory.

The second stage of laboratory research involved longer 3DSensor specimens up to 8 m, which were deformed considering static schemes (including simply supported beam and continuous beam). For reference measurements, vibrating wire (VW) displacement sensors were chosen with an accuracy of 0.1% of their full scale. The procedure was the same as in the research presented in Section 3.1; however, the measurement station in the form of stiff steel framework was applied to control the experiment—Figure 17a,b.

**Figure 18:**
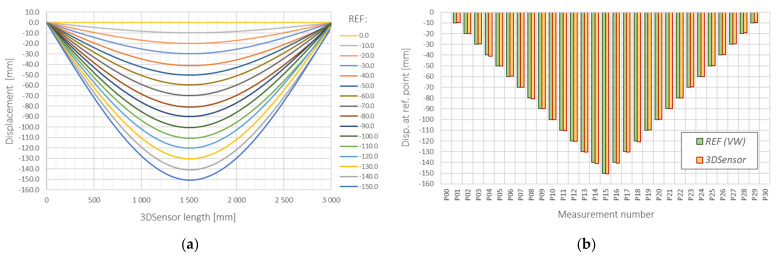
3DSensor in the scheme of simply supported beam: (**a**) calculated vertical displacements; (**b**) comparison between calculated midspan displacements with a reference vibrating wire sensor.

The example results for a scheme of a simply supported beam in the form of vertical displacement profiles are shown in Figure 1a for subsequent load steps. The applied spatial resolution was equal to 10 mm. Comparison with the independent VW technique is presented in Figure 18b, indicating very good consistency: the mean absolute and relative errors were equal to 0.055 mm and 0.420% with corresponding standard deviations of 0.494 mm and 0.793%. A reference gauge was installed in the midspan of the 3DSensor. The maximum value of vertical displacement in this point analysed during research was equal to 150 mm.

**Figure 19:**
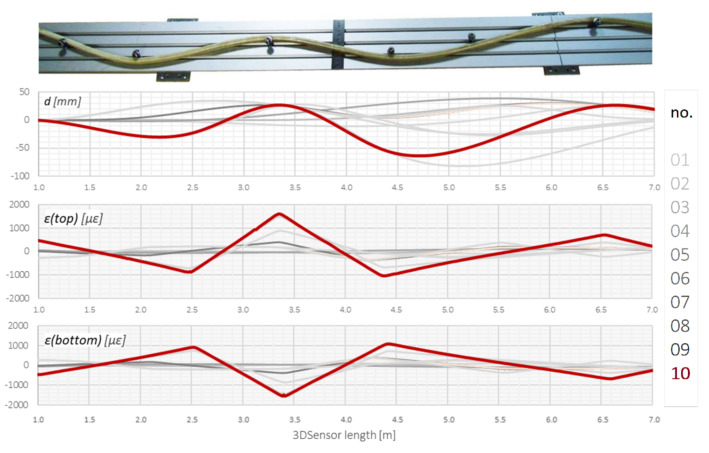
Example strain (top and bottom) and displacement data for continuous beam static scheme during subsequent configurations (01–10).

Another example is related to a continuous scheme, where 3DSensor was deformed using an aluminium strip with pins. Displacements were verified by inductive LVDT sensors with an accuracy better than ±0.05% of their full scale. Figure 19 shows the deformed specimen and corresponding calculated displacements *d*, as well as raw strain data for both the bottom and top surface of the sensor’s core.

**Figure 20:**
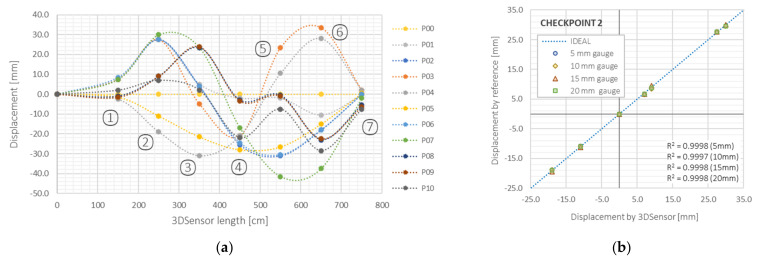
3DSensor in the scheme of continuous beam: (**a**) example displacements profiles with checkpoint reference values (1–7); (**b**) comparison between 3DSensor and reference displacements at checkpoint no. 2.

Error analysis was performed in seven checkpoints at locations of reference sensors, shown in Figure 20a. Displacement profiles were calculated for different configurations (dashed lines) and compared to LVDT results (dots), indicating very good compliance. The mean relative errors were of the same order as in the previous studies on cantilever and simply supported schemes (less than 0.5%). What is more, the influence of spatial resolution (gauge base) was investigated during data postprocessing. Displacement profiles were calculated based on strains presented with different spatial resolutions (5, 10, 15 and 20 mm) and each time, the obtained differences were negligible. Figure 20b shows the comparison of 3DSensor results against reference results with almost 100% value of determination coefficient of determination *R*^2^.

## 4. In Situ Application inside Embankment

### 4.1. General Description

The construction of embankments is a challenging process requiring strict control, especially in settlements due to ground compaction. Theoretical predictions, including even the most advanced calculations in three-dimensional FE models, are subjected to considerable uncertainties. Thus, gathering reliable measurement data related to the technical condition of the structure is of great importance. They allow for the improvement of work quality, but also reduce construction time and thereby save money. The purpose of this section is to present the practical application of the new distributed fibre optic 3DSensor, installed in field conditions to monitor vertical displacements under embankment during its construction.

**Figure 21:**
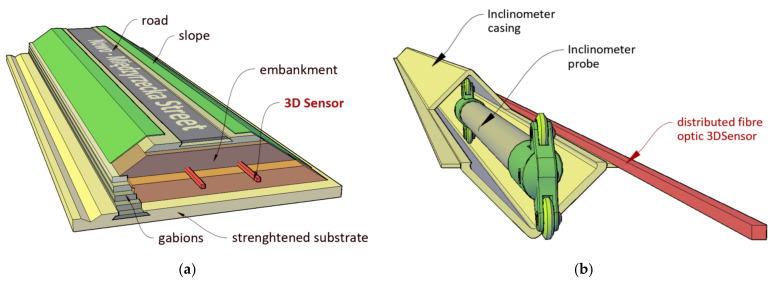
(**a**) Spatial visualization of monitored embankment with 2 lines of 3DSensor; (**b**) spatial visualization of 3DSensor with reference, longitudinal inclinometer system.

The investment was carried out in Bielsko-Biała (southern Poland). The 48 m long monitoring section was located next to the abutment of a bridge. It was equipped with two longitudinal 3DSenors (Figure 21a), but also with transverse and longitudinal inclinometers (Figure 21b), which were applied as a reference technique to assess the accuracy and performance of the new DFOS displacement sensor.

Inclinometer systems are commonly used in geotechnical applications [49,50]. Based on angle measurements (probe rotation against vertical direction determined by gravity) in selected positions over length, it is possible to calculate displacements profile [51]. In the considered project, spatial resolution for inclinometer measurements was equal to 0.5 m. The technical specifications of the probe [52] are summarized in Table 4.

### 4.2. Sensors Delivery, Location and Installation

**Figure 22:**
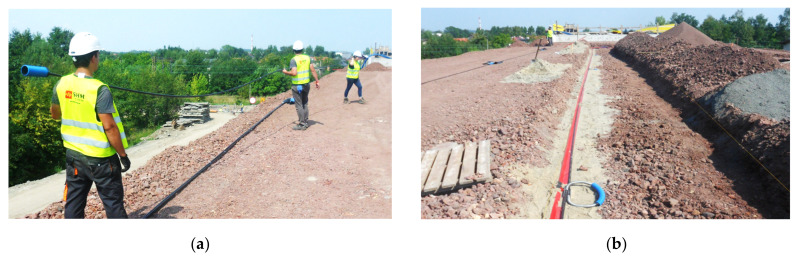
(**a**) The view of 3DSensor just before installation in the embankment; (**b**) the view of ready measurement line with 3DSensor and inclinometer casing.

The 3DSensors were delivered on site with 12 m long sections (Figure 22a) and then they were connected on site (Figure 22b). One of the challenges of the project was to verify the connection between the sensor’s segments (see also line 9 in Table 1). The connection was designed so as not to impact the measuring length. For particular sections that were overlapping, an appropriate algorithm was applied to compensate for this fact during data postprocessing. However, the production technology (pultrusion) is a continuous process and thus sensors can be delivered on site at any lengths.

Two independent lines (A and B) of 3DSensors were installed along the 48 m section with the spacing of approximately 200 cm. Along the section B, the longitudinal inclinometer was installed as a reference technique. To analyse embankment deformation in a complex way, the entire system was also equipped with:

- Two transverse inclinometers;
- Four spot tiltmeters for rotations analysis at start and end points of the measuring lines;
- Geodetic benchmarks located in technical wells to analyse deformations in reference to the global coordinate system.

**Figure 23:**
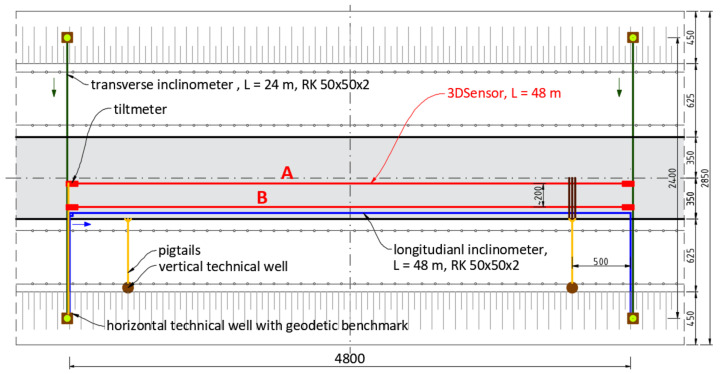
Plane view of the monitored embankment section (cm).

**Figure 24:**
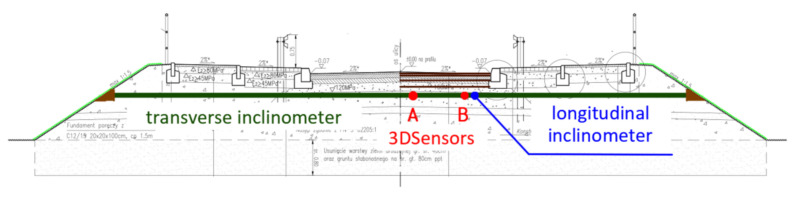
Cross-section of the embankment at the location of transverse inclinometer.

**Figure 25:**
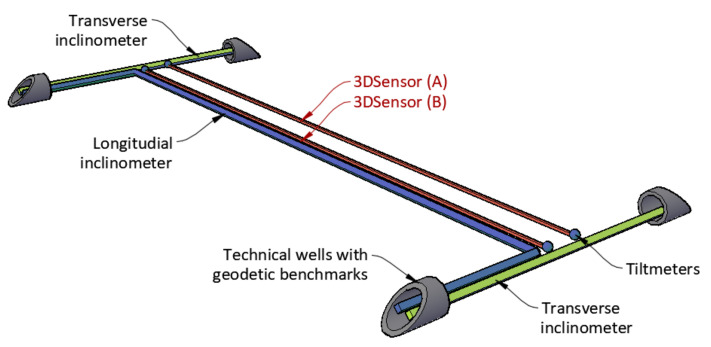
Spatial visualization of the monitoring system with 3DSensors, inclinometers and technical wells.

The plan view of the monitored part of embankment is shown in Figure 23, characteristic cross-section over transverse inclinometer in Figure 24 and spatial visualization of the entire monitoring system in Figure 25.

All sensors were installed in the groves filled with fine-grained sand to avoid local pressures caused by the coarse aggregate used for embankment construction. Furthermore, the entire measurement lines were protected from the top with a geotextile. All the optical pigtails and signal cables from applied spot tiltmeters were led to the technical wells located at the embankment slope, allowing for connection during periodical measurement session. Distributed fibre optic 3DSensor along the section B was tied to the reference inclinometer casing to ensure the correct comparison between measured vertical displacement profiles.

### 4.3. Example Results and Discussion

Measurements using distributed fibre optic strain or displacement sensors are always made in reference to a zero reading. In the considered project, this reading was taken after stabilization of the sensor by the aggregate layers with the thickness of approximately 100 cm. The next measurement session took place during construction, but measurements will be also performed during the long-term operation of the road embankment. The example results described further refer to the stage when the embankment was completed—just before it was put into operation.

**Figure 26:**
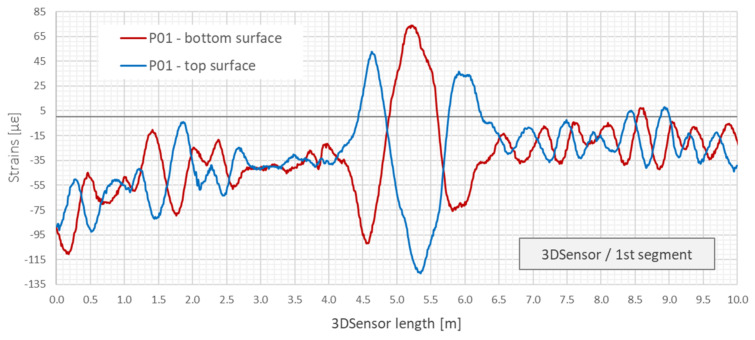
Strain data from the bottom and top surface of 3DSensor in length domain (only first 10 m were selected for clear presentation).

Data from distributed fibre optic displacement sensor were analysed with 10 mm spatial resolution, which means 100 measuring gauges along 1 m of 3DSensor. Analysis was only focused on vertical displacements. The obtained raw strain data were averaged from two bottom fibres and two top fibres to increase the final accuracy, and they are presented in Figure 26. Only the first 10 m of the sensor is shown to present the operation rule and keep appropriate clarity of the drawing. It can be noticed that the sensor was subjected both to the bending and axial actions related to force and temperature changes. However, only bending effects are responsible for changing the shape of the sensor.

**Figure 27:**
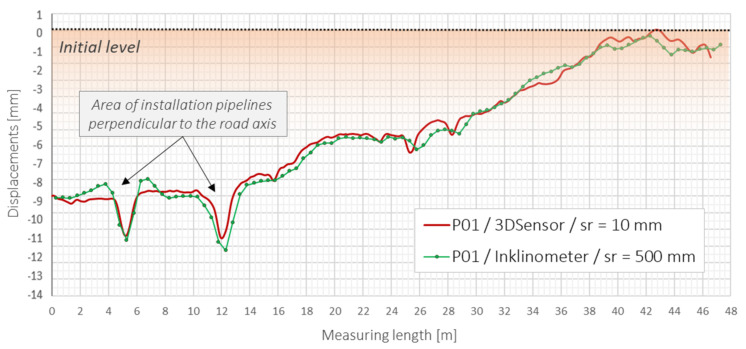
Displacements profiles measured by 3DSensor (B) and reference longitudinal inclinometer over entire measurement length of 48 m.

The averaged strain data were then used to calculate the displacement profile using the trapezoidal method described in Section 2 of this article. Thanks to the thermal self-compensation approach, there was no need to perform additional temperature measurements for data correction. The obtained displacement profile was compared to that obtained from reference longitudinal inclinometer. Data are presented in Figure 27, showing very good compliance. The mean difference along the entire 48 m length between these two independent techniques was less than 0.1 mm with standard deviation less than 0.5 mm. It should be noticed that spatial resolution for distributed fibre optic sensing was in this case 50 times better in comparison with the inclinometer system. That it is why the DFOS approach can be treated as more reliable.

Analysing the above plots, local sinkholes can be observed at a location of approximately 5 and 12 m. The relative displacements are equal to about 2–3 mm over a length of few meters; however, they could be precisely detected by two applied measurement techniques. Based on the project documentation and schedule of construction work, they were identified as areas of installation pipelines perpendicular to the road axis. These are potentially dangerous regions where the ground has been compacted less than within the rest of the structure. Settlement observation will continue in order to analyse the behaviour of the entire embankment, with particular attention to critical areas.

### 4.4. Another Proved Applications–Brief Review

**Figure 28:**
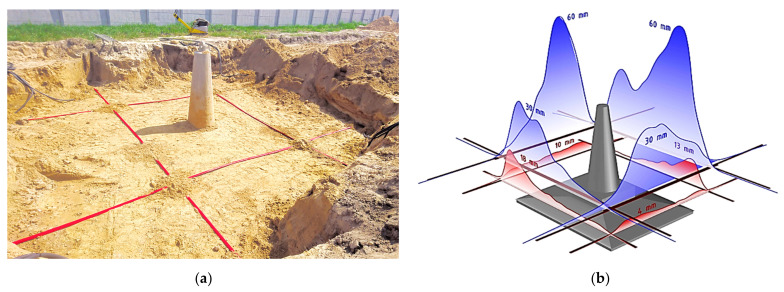
Geotechnical research field: (**a**) view of installed 3DSensor around the footing [46]; (**b**) spatial visualization of displacements profile calculated for a given load step during pulling the footing out of the ground [46].

**Figure 29:**
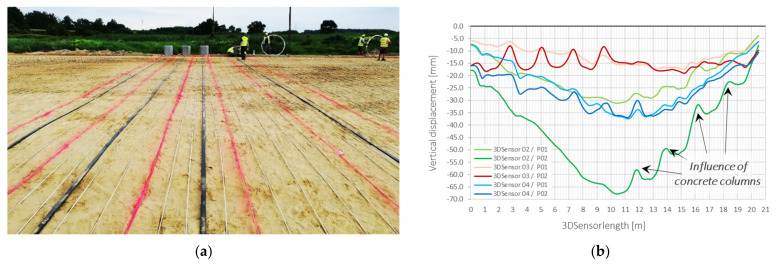
Embankment above the substrate strengthened with concrete columns: (**a**) 3DSensors’ installation; (**b**) example measured displacement profiles with visible influence of concrete columns.

**Figure 30:**
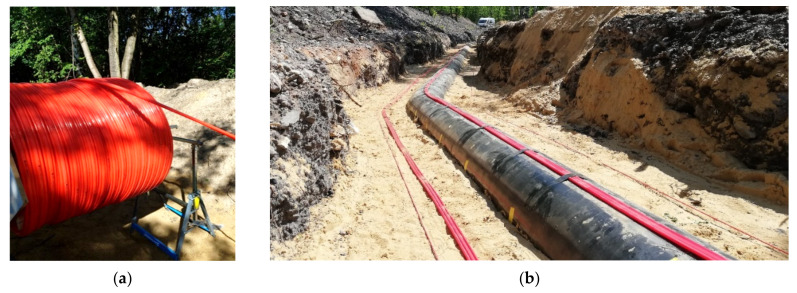
Gas pipeline equipped with 3DSensors: (**a**) delivery on site in the coil; (**b**) view of the installed sensors.

**Figure 31:**
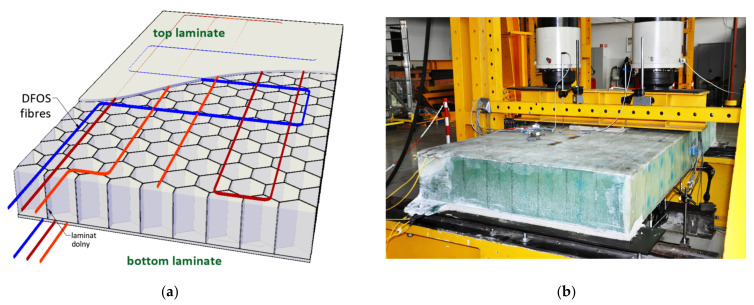
Idea of 3DSensor by integration of optical fibres during infusion of composite bridge panel: (**a**) designed spatial visualization; (**b**) ready panel before laboratory tests.

The performance of designed displacement 3DSensor was also checked within other geotechnical and engineering installation. This section gives just brief look on possible applications, which include:

1. Geotechnical research field [46], where different types of concrete footings design for electrical lines were pulled out from the ground for researching the shear plane. Figure 28a shows the installation process, while Figure 28b shows an example spatial visualization of displacement profiles obtained at a given load step;
2. Embankment [46] constructed above the substrate strengthened with concrete columns. Figure 29a shows sensors during installation, while Figure 29b shows example results in the form of displacement profiles with the visible influence of concrete columns;
3. Gas pipeline. Sensors were delivered in coil (Figure 30a), unrolled and installed along the entire 180 m length at designed positions (Figure 30b);
4. Composite bridge panel [45], where during the infusion process, optical fibres were integrated with lower and upper laminates with the same idea as in the 3DSensor. Figure 31a shows the spatial visualization of designed panel, while in Figure 31b, the ready structure just before laboratory investigation is shown.

## 5. Conclusions

This article presents an innovative approach for structural health monitoring, based on the application of the new distributed fibre optic 3DSensor for displacement measurements. The novelty of the presented solution includes:

1. Design of the sensor for measuring displacements in three directions (X, Y, Z);
2. New application in geotechnics and civil engineering;
3. Production technology (arrangement and integration of optical fibres within the sensor’s core during pultrusion);
4. New composite material used as a sensor core.

The article describes operating principles, including the thermal compensations system. The design and performance of the sensor was checked based on a wide range of laboratory tests and in situ installations, one of which was described in more detail. The designed sensor is resistant to harsh environmental conditions and could be successfully applied for monitoring of linear structures such as embankments, dams, pipelines, roads, highways, especially located around landslide or mining areas.

Distributed measurements of settlements inside the road embankment proved the sensors’ accuracy and performance. The installation was preceded by both theoretical (analytical and numerical) analysis and detailed laboratory tests. Based on the research, technical specifications of the 3DSensor were determined and are summarized in Table 5.

The presented solution is very promising in the context of long-term structural health monitoring, especially taking into account the dynamic development of distributed fibre optic sensing technologies. Research is now continuing within other field applications, and the lessons learned will allow for further improvements and wider use.

## 6. Patents

US patent, Application number: 15/849,804, Patent number: US 10,620,018 B2, Title: Method for measuring the displacement profile of buildings and sensor therefor, Application date: 21 December 2017, Publication date: 14 April 2020, Applicant: SHM System Sp. z o.o., Sp. kom., Inventors: Bednarski Ł., Sieńko R.

Polish patent (PL), Application number: P.412838, Patent number: Pat.235392, Title: Method for continuous measurement of the building objects relocation profile and a sensor for execution of this method, Application date: 24 June 2015, Publication date: 13 February 2020, Applicant: SHM System Sp. z o.o., Sp. kom., Inventors: Bednarski Ł., Sieńko R.

EP3314202A1, Title: Method for measuring the displacement profile of buildings and sensor therefor, Application number: EP16744907A, Application date: 17 June 2016, Publication number: EP3314202A1, Publication date: 2 May 2018, Applicant: SHM System Sp. z o.o., Sp. kom., Inventors: Bednarski Ł., Sieńko R.

Canadian Intellectual Property Office, Patent Application: CA 2989301, Title: Method for measuring the displacement profile of buildings and sensor therefor, PCT Filling date: 17 June 2016, Open to public inspection: 29 December 2016, Applicant: SHM System Sp. z o.o., Sp. kom., Inventors: Bednarski Ł., Sieńko R.

International Publication Number: WO 2016/209099 A1, Title: Method for measuring the displacement profile of buildings and sensor therefor, Publication date: 29 December 2016, Applicant: SHM System Sp. z o.o., Sp. kom., Inventors: Bednarski Ł., Sieńko R.

## Figures and Tables

**Table 1:** Issues addressed during the development of new distributed fibre optic displacement sensor.

No.	Issue	Work Description	Solution/Result
1	Selection and calibration of optical fibre and its primary coating.	Statistical laboratory research on selected optical fibres with different types of primary coatings (acrylate, polyimide, experimental sand-grained).	Selection of the standard telecom SM9/125 optical fibre in soft acrylate coating.
2	Analysis of the influence of additional (secondary) coatings.	Statistical laboratory research on selected types of secondary coatings, including tight jackets.	Removal of all additional fibre coatings.
3	Selection of the material for sensor’s core ensuring high elastic range.	Statistical laboratory research on selected materials (steel, plastics, rubbers, composites).	Composite core with elastic strain range up to ±4%.
4	Integration method of the fibres and sensor’s core.	Research on different types of adhesives and other technological possibilities.	Integration of the fibres at production (pultrusion) stage.
5	Algorithms for data processing.	Theoretical studies including state-of-the-art review, analytical work and numerical simulations.	4 algorithms for displacement calculations: beam deflection equation, explicit methods of the first and the second order, trapezoidal method.
6	Thermal compensation.	Laboratory research on thermal compensation systems effectiveness.	Additional compensating fibre, thermal self-compensation system.
7	Accuracy of displacements.	Statistical laboratory research on different versions of the sensor with reference techniques.	Data sheet including technical specifications.
8	Repeatability and long-term stability.	Statistical laboratory research over long term.	Data sheet including technical specifications.
9	Connection of the sensor’s segments.	Laboratory research on different types of connections and protective casings.	Design of the way to connect the sensor in case of breakage.
10	Wide range of proven field applications.	Sensors’ demonstration installations in field conditions (2 x bridge, 1 x industrial tower, 5 x geotechnical structures, inc. slurry walls and embankments, others).	Installation, measurements, data processing, real performance → lessons learned (see also Section 4).

**Table 2:** Selected parameters of applied optical backscatter reflectometer.

Parameter	Value	Unit
Measurement length (standard mode)	70	m
Spatial resolution (gauge spacing) ^1^	10	mm
Gauge length ^2^	10	mm
Strain measurement resolution	±1.0	mm

^1^ Recommended spatial resolution. ^2^ Recommended length of the gauge base.

**Table 3:** Example results from cantilever 3DSensor with error analysis.

No.	Vertical Displacements				Horizontal Shortenings over *x* Axis			
	d_z,ref_ (mm)	dz_,3D_ (mm)	e_absolute_ (mm)	e_relative_ (%)	d_z,FEA_ (mm)	dx_,3DS_ (mm)	e_absolute_ (mm)	e_relative_ (%)
P01	3.000	2.994	0.006	0.196	0.013	0.013	0.000	1.831
P02	6.001	6.017	−0.016	−0.268	0.054	0.051	0.003	4.696
P03	9.000	9.019	−0.019	−0.208	0.121	0.116	0.005	4.461
P04	12.001	12.049	−0.048	−0.397	0.214	0.206	0.008	3.648
P05	15.000	15.054	−0.054	−0.362	0.335	0.322	0.013	3.962
P06	17.999	18.069	−0.070	−0.387	0.482	0.463	0.019	3.899
P07	21.000	21.084	−0.084	−0.401	0.656	0.631	0.025	3.880
P08	23.999	24.078	−0.079	−0.330	0.857	0.822	0.035	4.071
P09	27.001	27.037	−0.036	−0.133	1.085	1.033	0.052	4.779
P10	30.000	30.218	−0.218	−0.727	1.339	1.290	0.049	3.677
P11	33.000	33.159	−0.159	−0.483	1.620	1.555	0.065	4.040
P12	36.001	36.108	−0.107	−0.296	1.928	1.844	0.084	4.342
P13	39.000	39.101	−0.101	−0.258	2.263	2.164	0.099	4.396
P14	42.002	42.376	−0.374	−0.891	2.624	2.535	0.089	3.390
P15	39.001	38.953	0.048	0.123	2.263	2.142	0.121	5.328
P16	36.001	36.021	−0.020	−0.055	1.928	1.829	0.099	5.122
P17	33.001	33.050	−0.049	−0.148	1.62	1.538	0.082	5.059
P18	30.001	30.055	−0.054	−0.181	1.339	1.270	0.069	5.137
P19	27.000	27.078	−0.078	−0.289	1.085	1.029	0.056	5.117
P20	24.000	24.084	−0.084	−0.348	0.857	0.813	0.044	5.125
P21	21.000	21.071	−0.071	−0.337	0.656	0.621	0.035	5.286
P22	18.000	18.079	−0.079	−0.438	0.482	0.456	0.026	5.358
P23	15.000	15.070	−0.070	−0.464	0.335	0.316	0.019	5.571
P24	12.000	12.064	−0.064	−0.535	0.214	0.202	0.012	5.715
P25	9.000	9.062	−0.062	−0.687	0.121	0.113	0.008	6.530
P26	6.001	6.069	−0.068	−1.133	0.054	0.050	0.004	7.374
P27	3.001	3.063	−0.062	−2.080	0.013	0.012	0.001	5.292
		Mean:	−0.077	−0.427		Mean:	0.042	4.707
		Stdv ^1^:	0.076	0.424		Stdv ^1^:	0.035	1.049

^1^ Standard deviation.

**Table 4:** Technical specifications of applied reference inclinometer system [53].

Parameter	Value
Displacement range	±250 mm/m
Displacement resolution	±3 mm/30 m
Displacement resolution	±0.02 mm/m
Spatial resolution	500–1000 mm
Type of the probe	MEMS
Operation temperature	from −40 °C to +85 °C

**Table 5:** Technical specifications of distributed fibre optic displacement 3DSensor [53].

Parameter	Value
Displacement range	any, dependent on static scheme
Displacement resolution	±1 mm
Operation temperature	from −20 °C to +60 °C
Standard dimensions of active core ^1^	8 × 6 mm
Standard external dimensions ^1^	50 × 15 mm
Sensor material	PLFRP + PE
Light scattering ^2^	Rayleigh, Brillouin, Raman
Delivery method	coils or straight sections
Length	any length made to order

^1^ Dimension can be adjusted depending on specific requirements. ^2^ Compatible with devices based on such phenomena.

## Data Availability

Not applicable.
